# Temporary cable force monitoring techniques during bridge construction-phase: the Tajo River Viaduct experience

**DOI:** 10.1038/s41598-022-11746-z

**Published:** 2022-05-11

**Authors:** Alvaro Gaute-Alonso, David Garcia-Sanchez, Carlos Alonso-Cobo, Iñigo Calderon-Uriszar-Aldaca

**Affiliations:** 1 Grupo de Instrumentación y Análisis Dinámico de Estructuras, University of Cantabria, Santander, Spain; 2 TECNALIA Basque Research and Technology Alliance (BRTA), Derio, Spain; 3 Structural and Mechanical Engineering Area, University of Cantabria, Santander, Spain; 4 University of Navarra, Pamplona, Spain

**Keywords:** Engineering, Civil engineering

## Abstract

This article deals with the comparative analysis of current cable force monitoring techniques. In addition, the experience of three cable stress monitoring techniques during the construction phase is included: (a) the installation of load cells on the active anchorages of the cables, (b) the installation of unidirectional strain gauges, and (c) the evaluation of stresses in cables applying the vibrating wire technique by means of the installation of accelerometers. The main advantages and disadvantages of each technique analysed are highlighted in the Construction Process context of the Tajo Viaduct, one of the most singular viaducts recently built in Spain.

## Introduction

One of the solutions proposed by civil engineers to overcome large spans is to use cable-stayed or suspension bridges. The critical component of these structures lies in the vulnerability of the cables to problems or damage associated with fatigue and/or corrosion^1^ caused by dynamic loads such as cyclic traffic loads, wind loads, and other operational loads as well as environmental effects^2^. Structural Health Monitoring Systems (SHMS) are a very useful tool for the maintenance of structures. One of the main phases of SHMS design is to identify the parameters that define the behavior of structures^3,4^. Temporary suspension cables for constructive phase present the same problems and it is at this phase that the article looks in more detail at.

The most important parameter for the assessment of stress, and fatigue and corrosion damage of cables in service is the historical record of the axial stress over time of these structural elements. This parameter has been recognized as a useful indicator of damage by safety condition of stay cables and also suspension cables in bridges^5^, and real-time monitoring of this indicator has become essential for the assessment of possible fatigue damage in these structural elements. For this reason, monitoring and assessment of structural performance has become standard practice to ensure the safety and durability of cable-stayed or suspended structures^6–9^.

## Assessing safety conditions of construction cables by direct and indirect techniques

Several types non-destructive testing are used to diagnose the safe condition of stay cables in bridges^9^, such as ultrasonic testing, the magnetic flux leakage detection technique^10^, or X-rays. Although effective, these techniques are more suitable for the assessment of out-of-service stays.

On the one hand, various devices have been developed for the direct measurement of strain in bridge cables, such as load cells^9–15^, optical fibre Bragg grating sensors^16^, or elastomagnetic strain sensors^17,18^.

These sensors are able, thanks to their specific technologies, to accurately determine the stress experienced by the cable, and, when connected to a Structural Monitoring System (SMS), it is possible to create a long-term historical record of cable stresses as well as to access this data in real time from any remote location.

On the other hand, the most common indirect methodology for the rapid assessment of bridge cable stresses is the vibrating wire technique. This method is based on the relationship between cable stress and its vibration frequency, which can be correctly identified from the recording of accelerations during free vibration of suspension cables^19–24^. The application of this method requires the use of spectral decomposition techniques that allow for a real-time determination of the stresses in the suspension cables in bridges by identifying the vibration frequencies during a free vibration regime^25–31^.

There are many types of sensors for each of the measurement techniques^32–35^.

### Electronical and micro electro-mechanical system sensors (MEMS)

Electronical sensors transform the measurement (parameter to be measured) into a change in voltage, current, resistance, capacitance or inductance. Electrical sensors come in many varieties and are often very simple to interface to any datalogger system. Examples include potentiometer displacement sensors, resistive strain-gauges, load cells, MEMS^36^ tiltmeters and piezoelectric sensors. Electrical sensors are often relatively inexpensive but can be subject to drift and can be affected or damaged by electromagnetic disturbances such as power lines.

### Vibrating wire

Vibrating Wire (VW) sensors^37^ transform the measuring into a chance in the vibration frequency of a wire. In the case of VW strain sensor, a change in strain results in a change in the tension of a steel wire, while in the case of VW piezometers the deformation of a membrane due to a change in water pressure also induces a change in the tension of the sensor wire. Once the wire is excited by an electromagnet, it is possible to accurately measure its vibration frequency. The frequency measurements are very accurate and stable, which is why these sensors have become a standard for accurate long-term measurement in geotechnical and structural monitoring.

### Optical fibre sensors

From many points of view, optical fibre sensors^38^ are the ideal transducers for structural health monitoring. These sensors transform the quantities to be measured into a change in the propagation characteristic of the light travelling through the optical fibre. Being durable, stable and insensitive to external disturbances, they are particularly useful for long-term health assessment of civil structures and geostructures. Many different fibre optic sensor technologies exist, including Fibre Bragg Gratings, SOFO Interferometer, Fabry Perot Interferometer and distributes Brillouin and Raman sensors, and offer a wide range of performances and suitability for different applications.

In the early 1990s, fibre optic sensors made a major entry into the sensor industry and now have an established presence in the structural sensing industry^39^.

### Distributed sensing

Distributed fibre sensors^40,41^ represent a paradigm shift in terms of monitoring and sensing. Distributed sensors are able to detect at any point along a single standard telecommunication fibre optics, which allows discriminating different positions of the measured parameter along the fibre, transferring strain and temperature from the structure to the fibre.

Distributed sensors are specially recommended^42^ for detecting and discriminating events at any point within a structure.

### Optical/radar/laser

These techniques are particularly useful for monitoring the global movements of structures^43^. They include traditional geodesy methods, total stations with or without target prisms installed on the structure, laser distance meters and laser profilometers as well as terrestrial radars (including synthetic aperture radar). Those techniques can be sued for example, to measure the deformation of a bridge, the movement of a tower, the progression of a landslide or the deflection of a dam.

### Image based techniques

Olaszek^44^ developed a method that incorporated the photogrammetric principle with computer vision technique to investigate slow dynamic characteristics of bridges.

Patsias and Staszewski^45^ and Yoshida et al.^46^ started the use of videogrammetric techniques to measure mode shapes of a beam and to capture the 3D dynamic behavior of different structures. Chung et al.^47^ used digital image techniques for identifying nonlinear characteristics in more complex structural systems. Chang and Ji^48^ developed a two-camera videogrammetric technique for measuring a 3D structural vibration response at laboratory level. In^49^, Ji and Chang proposed a novel nontarget technique based on image analysis using one digital camera for cable vibration measurement but, again, it is a proof-of-concept.

These techniques, although promising and most of them verified at laboratory level or under controlled conditions, do not seem to provide complete dynamic information, only a part of the free vibration. Therefore, application during construction must await a more advanced level of development.

## Application in the construction process of the Tajo River Viaduct

### General Tajo River Viaduct description

The Tajo River Viaduct belongs to the Madrid–Extremadura high-speed rail. It is located in the province of Caceres and has a total length of 1488 m. Its span distribution is determined by the width of the Tajo River, over which the arched viaduct spans 324 m. The above deck is divided into six spans of 54 m each. The access spans are 60 m long, with two 57 m transition spans between them and the deck spans above the arch. The results in an appropriate and harmonious distribution of the 26 spans that make up the Tajo River Viaduct deck: 45 m + 9 × 60 m + 57 m + 6 × 54 m + 57 m + 7 × 60 m + 45 m^50,51^.

The layout of the viaduct presents a very wide curve and then a straight line throughout the rest of the bridge. The most emblematic element of this viaduct is the arch, with a span of 324 m between its supports, which rises above its foundations to a height of 70 m and above the maximum level of the Alcántara Reservoir to a height of over 80 m.

### Construction Process

**Figure 1:**
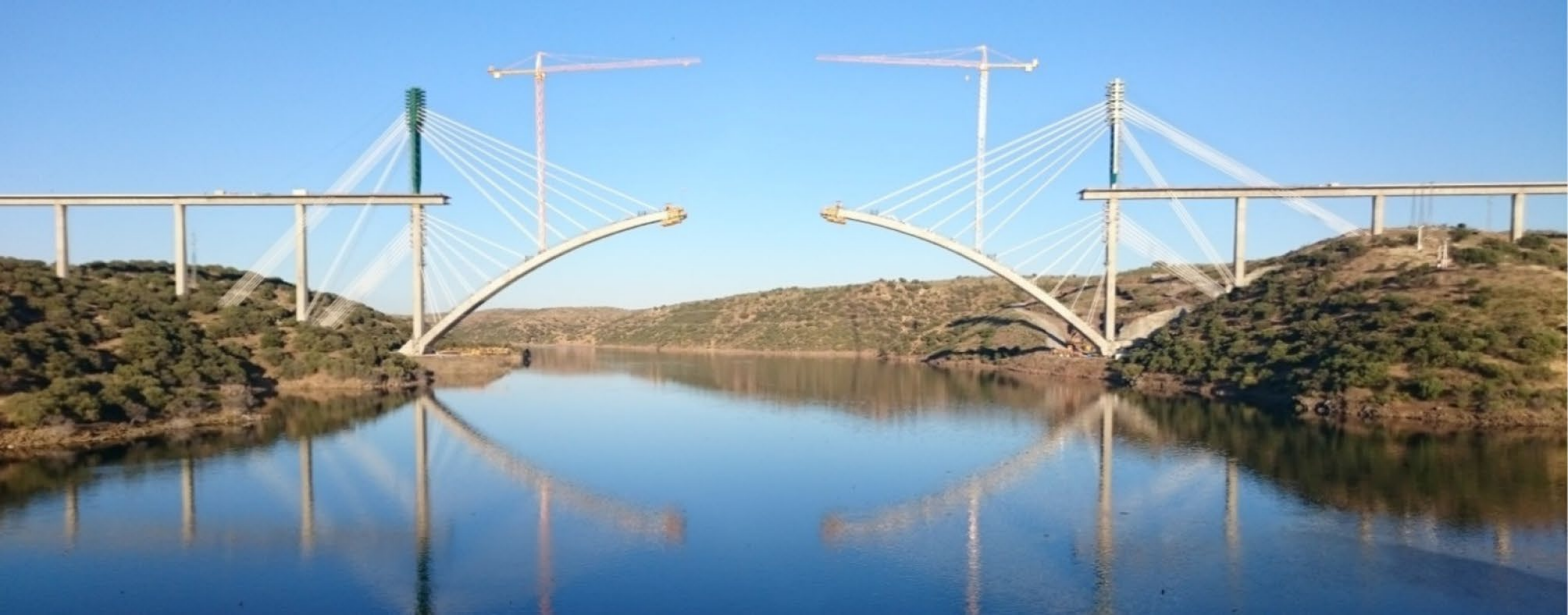
Tajo River Viaduct during its construction.

The arch was built using the technique of successive cable-stayed cantilevers, with two temporary towers placed on the piers on both banks of the river (see Fig. 1). Each tower was braced to the foundations of the neighboring piers, so it was necessary to provide these foundations with the prestressed ground anchorage units. For the arch construction phase, fifteen pairs of cables supported each half full arch, and a further fifteen pairs supported the tower. The length of these cables ranged from 80 to 180 m.

Each half arch is made up of a total of 46 segments, each of 4 m long. The concreting trolley is a metallic element that supports the formwork of each segment and its concreting. This trolley was placed in the area of the arch that had been concreted most recently, in order to prepare for the concreting of the next segment.

Once the arch was completed, the temporary cable system consisting of the temporary towers, cables and ground anchors was dismantled. At this point, two adjacent piers were built on either side of the arch which, together with the segmental, support the deck above the arch. The deck was built span by span using self-supporting formwork from both abutments. To avoid creating excessive stresses in the arch, the deck was concreted symmetrically and allowed for a maximum displacement of only one span.

### Analysis of cable monitoring methodologies during bridge construction processes

During the structural monitoring of Tajo River Viaduct, three methodologies were used to monitor the tension in the temporary suspension cables of the main span arch-type: (a) strain gauge installed in the active anchorage of the temporary cables, (b) unidirectional strain gauges installed in one of the seven wires belonging to one of the cable strands, and (c) cable instrumentation by means of a unidirectional accelerometer.

#### Direct monitoring of the cables with strain-gauge load cells

**Figure 2:**
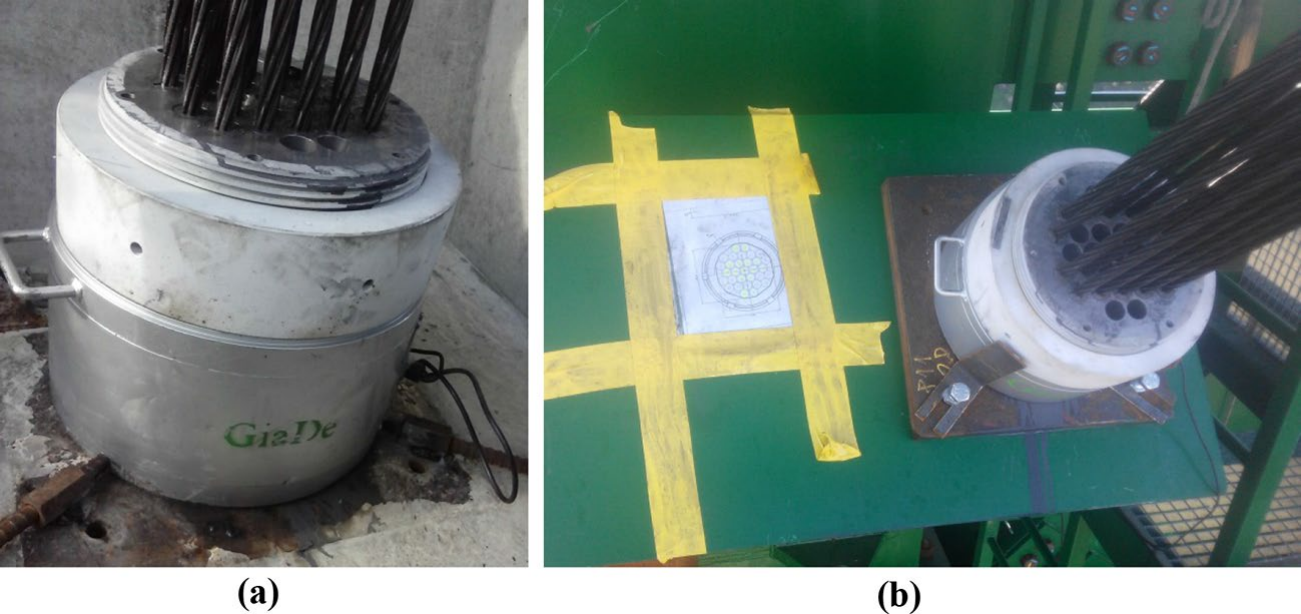
Tajo River Viaduct load cells: (**a**) pier load cell; (**b**) load cell in temporary stay tower.

The strain gauge load cells designed by the authors for the instrumentation of the temporary cables of the Tajo River Viaduct consisted of a metal ring between the cable anchor plate and the distribution plate on the pile or temporary tower. Due to the dimensions of the suspension cables, the average diameter of the load cells of the Tajo River Viaduct varied between 200 mm for the least loaded cables (2000 kN) and 500 mm for the most loaded cables (5500 kN) (see Fig. 2).

This technology makes it possible to determine the strain in the stay cable from the empirical characterization of the mean normal strain in the central ring of the load cell, see Eq. (1). For this purpose, the outer perimeter of the central ring is instrumented by the uniform distribution of bi-directional strain gauges connected in series by a full Wheatstone bridge configuration^52–55^.

1 $$F = \mathop{{\int\!\!\!\!\!\int}\mkern-21mu \bigcirc} {\sigma \cdot d{\Omega }} = \frac{{\mathop \sum \nolimits_{1}^{n} \varepsilon_{i} }}{n} \cdot E_{a} \cdot {\Omega }_{c}$$

where F = Stress in the suspension cable; σ = Normal stress; dΩ = Differential area at the central ring; ɛ_i_ = Normal strain at the ith strain gauge; n = Number of strain gauges; E_a_ = Modulus of elasticity of steel; Ω_c_ = Area of the central ring.

**Figure 3:**
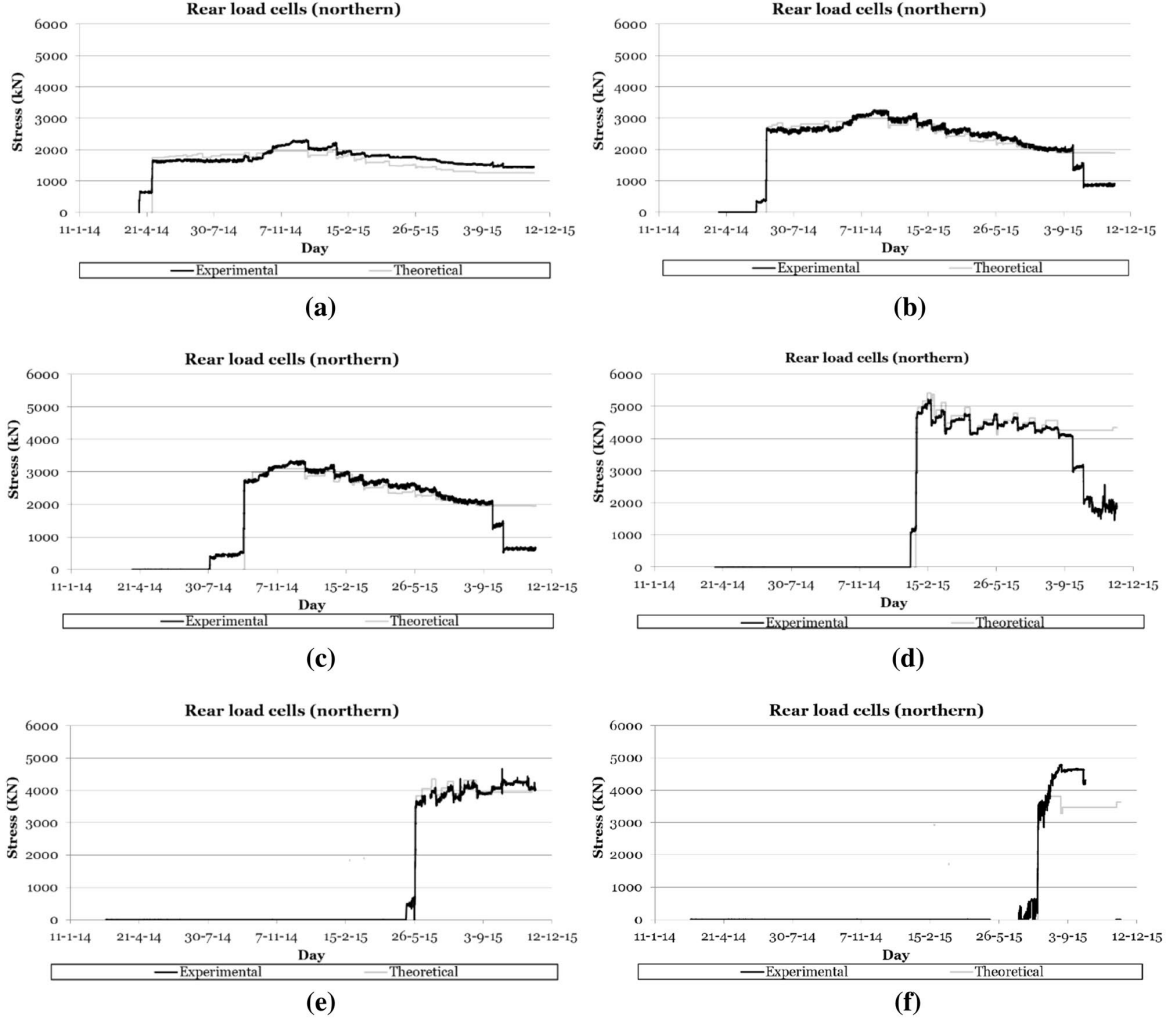
Recording the evolution of stresses in the retaining cables of the northern semi-arch: (**a**) 2nd cables family; (**b**) 4th cables family; (**c**) 6th cables family; (**d**) 8th cables family; (**e**) 12th cables family; (**f**) 14th cables family.

**Figure 4:**
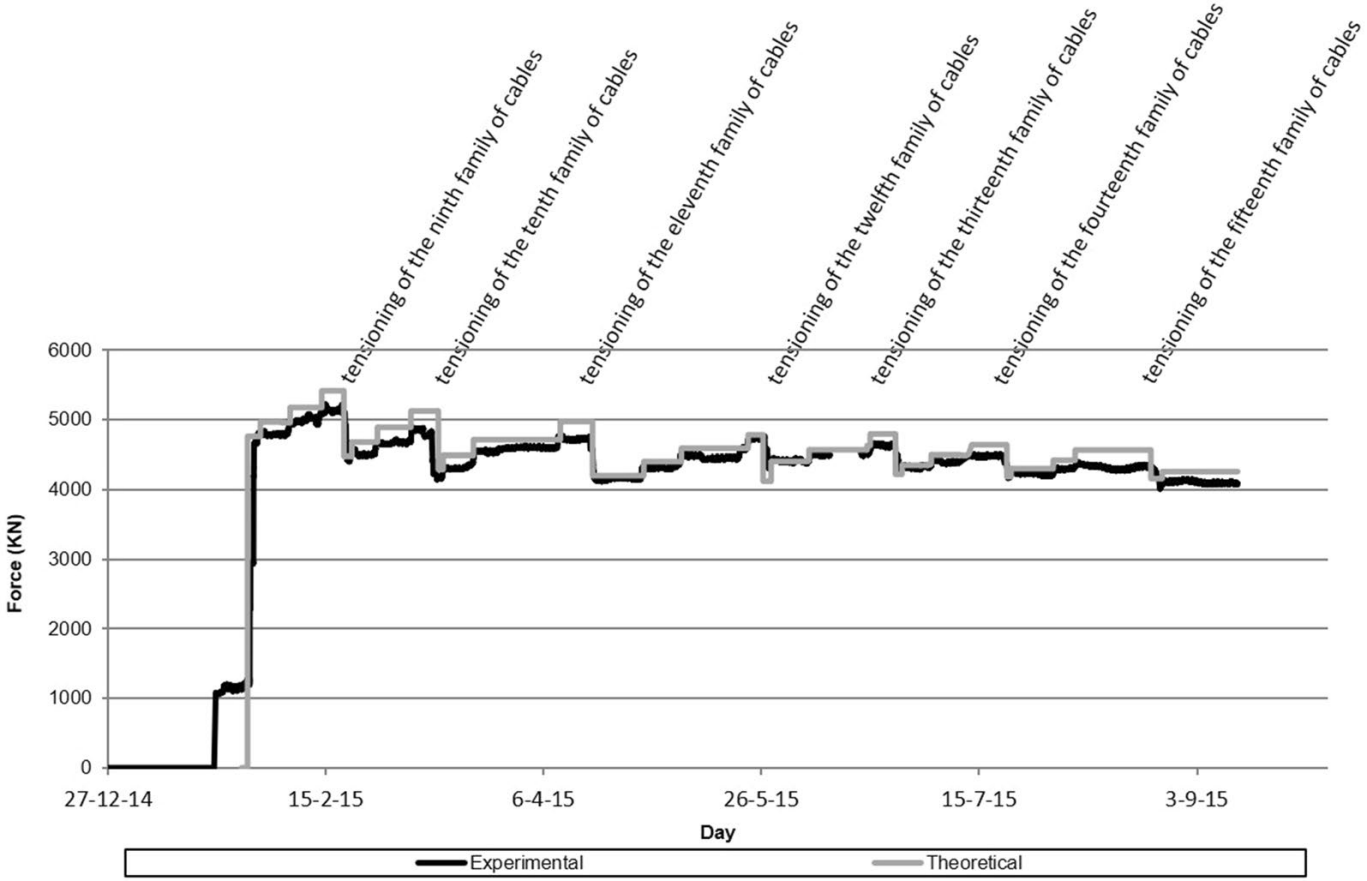
Evolution of the stress experienced by the eighth retaining cables family.

The load cells installed on the cables made it possible to certify the correct tensioning of the cable families and to characterise, in real time, the variations in the stresses experienced by the suspension cables during the different phases of the construction process (see Fig. 3). These devices made it possible to detect any of the following structural phenomenon occurring during the construction process: (1) the variation of stress in the cable due to the daily thermal increase, with stress increases of around 150 kN for daily temperature variations of 30 K (30 °C); (2) the stress variations due to the concreting of successive segments, with stress variations that could range from 200/300 kN in the cables closest to the concreted segment, to values lower than the daily variation in the cables furthest away; (3) the stress increases due to the tensioning of the successive families of cables (see Fig. 4), with stress variations greater than 500 kN in the families closest to the cables placed under load, and less than the daily variation of the load in the families furthest away; or (4) stress variations due to load readjustment operations in cables.

Figure 4 shows the evolution of the stress experienced by the stay cables of the eighth family. This graph shows the evolution of the stress characterized by the load cell installed in one of the rear stay cables, as well as the evolution of the theoretical stress predicted by the theoretical calculation model. The evolution of the stress experienced in the provisional stay cables of the eighth family, caused by the concreting of the successive arch segments can and the tensioning of the successive families of cables, can be clearly seen.

**Figure 5:**
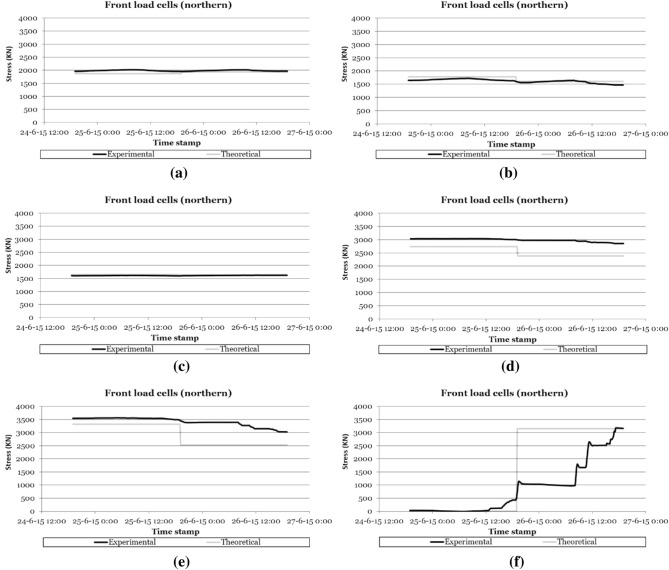
Recording the evolution of stresses in the stay cables of the northern semi-arch during tensioning of the thirteenth cable family: (**a**) 3rd cable family; (**b**) 5th cable family; (**c**) 7th cable family; (**d**) 9th cable family; (**e**) 11th cable family; (**f**) 13th cable family.

Figure 3 shows the evolution of the stress experienced by the different families of the retaining cables of the northern half arch and their comparison with the theoretical values of the project during all the construction phases of the main arch span, while Fig. 5 shows the evolution of the stresses in the suspension cables of the northern half arch during the loading of the cables corresponding to the 13^th^ family of temporary cables.

The values provided by the instrumentation of the provisional stays of the Tajo River Viaduct allowed the calculation models to be continuously updated with the reality of the construction site. The comparison and updating of the theoretical/empirical values made it possible to readjust the load values in the stay cables in the event of large deviations.

#### Direct monitoring of the cables by installing unidirectional strain gauges on a wire in the temporary cable

During the instrumentation of the cables corresponding to the first two families of temporary cables, the authors detected that the edge initially given to the strain gauge load cells was not sufficient to minimise the error induced by the irregularities in the boundary conditions. It was necessary to complement these sensors with other types of sensors to characterize the strain variations in the cables. It was decided to install two unidirectional strain gauges on one of the seven wires that make up one of the strands, connected together using a full Wheatstone bridge configuration^52–55^. This technology allows for the instrumentation of cables once loaded and enables the empirical characterization of the strain increases experienced by the cable.

**Figure 6:**
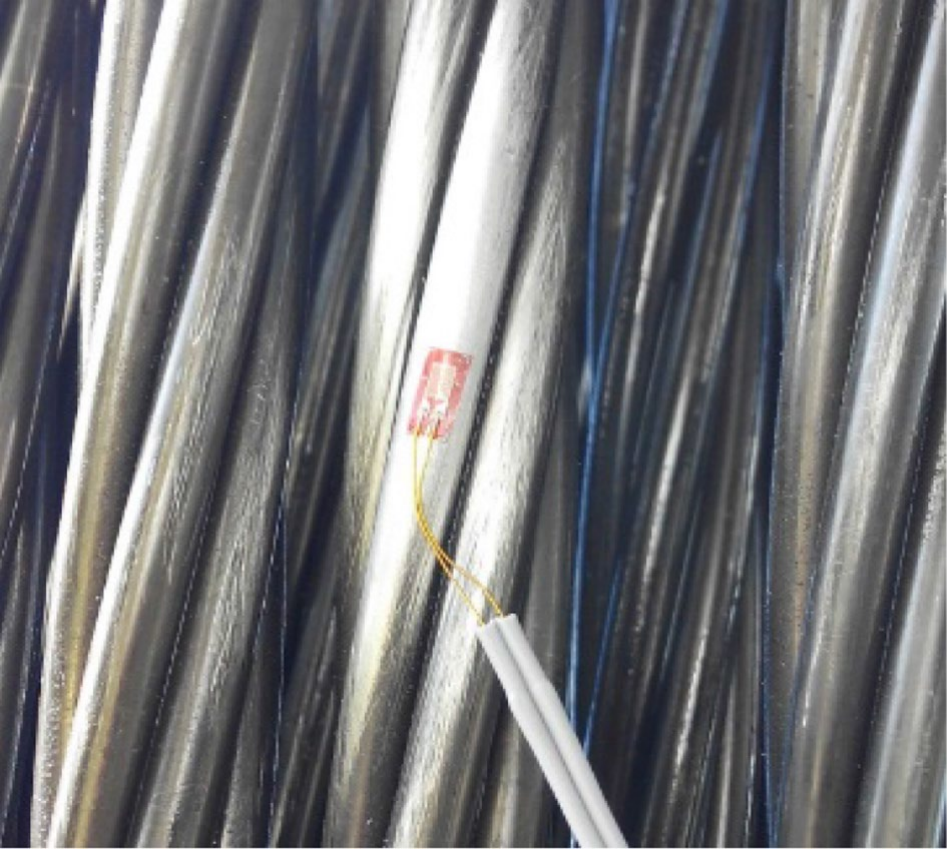
Installation of unidirectional strain gauges on one of the wires of a strand in a temporary cable.

**Figure 7:**
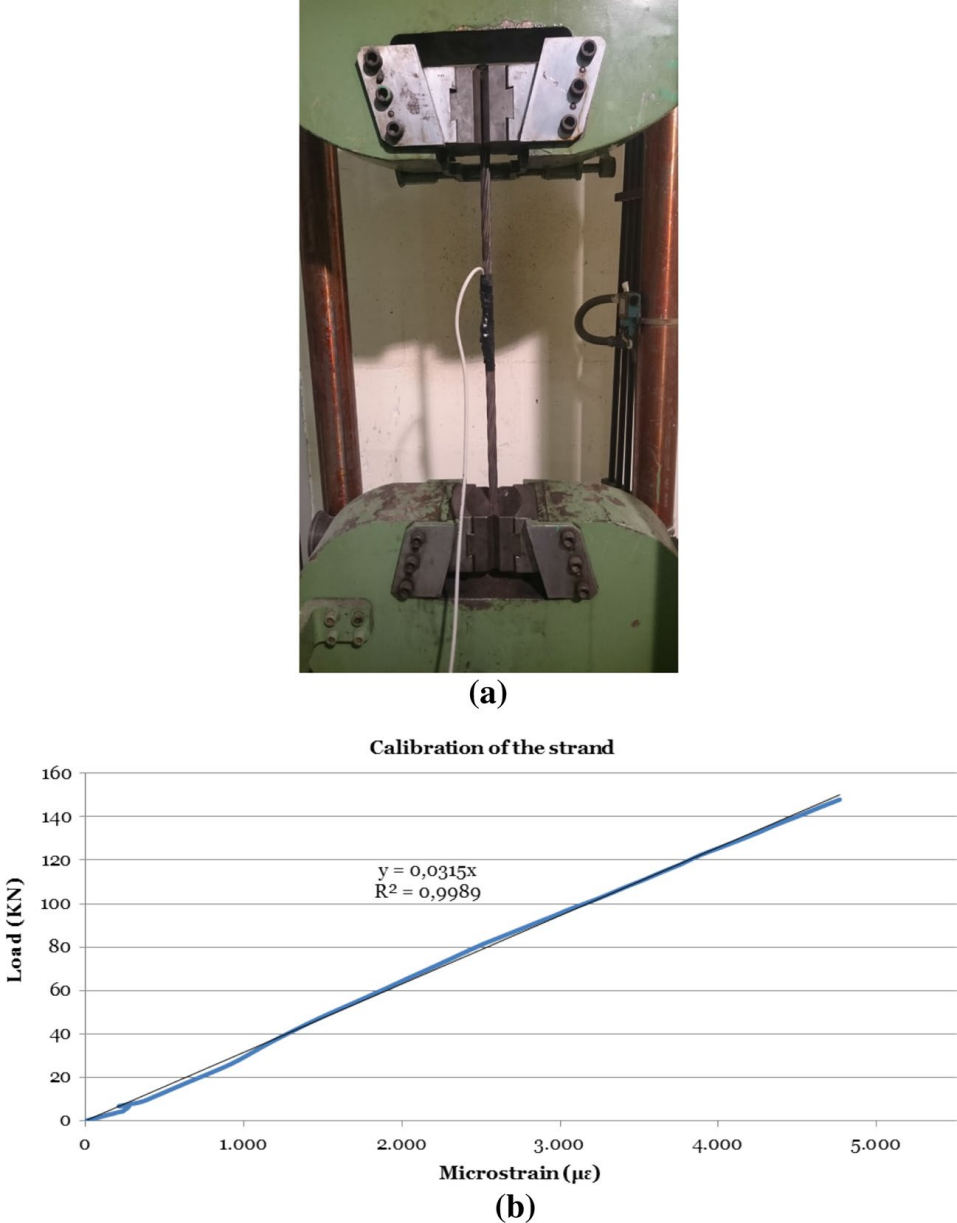
Calibration of the cable instrumentation method by the installation of unidirectional strain gauges: (**a**) prestressing strand in load-bearing gantry; (**b**) load/deflection curve during calibration test.

The steel wires that make up the strand are twisted together so that the directrix of these wires has a certain angular deviation with respect to the strand directrix (see Fig. 6). This fact makes the recovery of the strand deformation from the wire deformation indirect, and implies the need to perform the calibration of the measuring system in a tensiometer where the kN/µɛ correlation between strand tension and the wire deformation can be obtained (see Fig. 7). The tensioning of the provisional stay cables of the Tajo River Viaduct was carried out using the isotensioning technique^56^. This technique makes it possible to obtain identical tension in each of the strands that make up each stay cable. However, it is advisable to instrument several strands of the same stay cable in order to obtain an optimum measurement of the tension in the stay cable.

**Figure 8:**
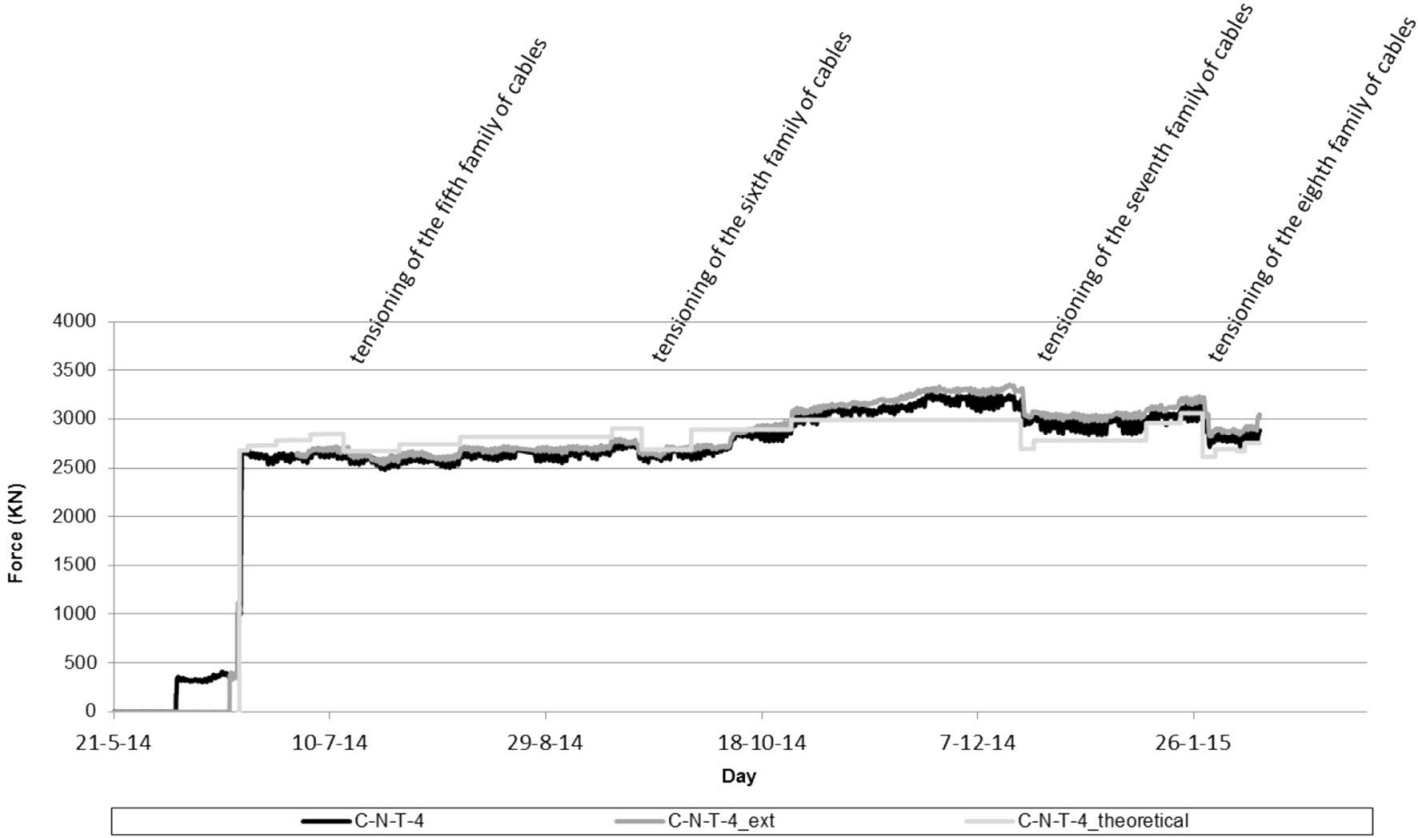
Evolution of the effort experienced by the fourth stay cable family.

The simultaneous instrumentation of the first two families of stay cables by means of load cells and unidirectional extensometers installed in one of the strands composing the stay cable provided a double contrast of the value of the stress value experienced by these structural elements. Figure 8 shows the evolution of the stress value in the instrumented stay cable of the fourth family provided by the load cell (CNT-4), by the extensometers (CNT-4_ext) and by the theoretical calculation model (CNT-4_theoretical). The graph shows the evolution of the stress in the stay cable due to the concreting of the successive arch segments and the stressing of the successive provisional stay cables.

#### Indirect monitoring of cables with unidirectional accelerometers

Due to the problems encountered in the measurements of the direct methodologies explained above for the first two families of cables of the Tajo River Viaduct, the authors proposed to carry out a weighing campaign of the cables belonging to these families using the vibrating wire technique. This technique makes it possible the measurement of the stress in bridge cables by characterising their vibration frequency and their mass^19,57,58^. The differential equation that relates the axial stress of a cable to its mass, its bending stiffness and its vibration frequency is as follows:

2 $$E \cdot I \cdot \frac{{\partial^{4} \vartheta \left( {x,t} \right)}}{{\partial x^{4} }} - T \cdot \frac{{\partial^{2} \vartheta \left( {x,t} \right)}}{{\partial x^{2} }} + m \cdot \frac{{\partial^{2} \vartheta \left( {x,t} \right)}}{{\partial t^{2} }} = 0$$

The solution Eq. (3) to the differential Eq. (2) allows for the axial stress of the cable to be obtained.

3 $$f = \frac{u}{2 \cdot L}\sqrt{\frac{T}{m}} \cdot \left[ {1 + 2 \cdot \sqrt {\frac{E \cdot I}{{F \cdot L^{2} }}} + \left( {4 + \frac{{u^{2} \cdot \pi^{2} }}{2}} \right) \cdot \frac{E \cdot I}{{F \cdot L^{2} }}} \right]$$

**Figure 9:**
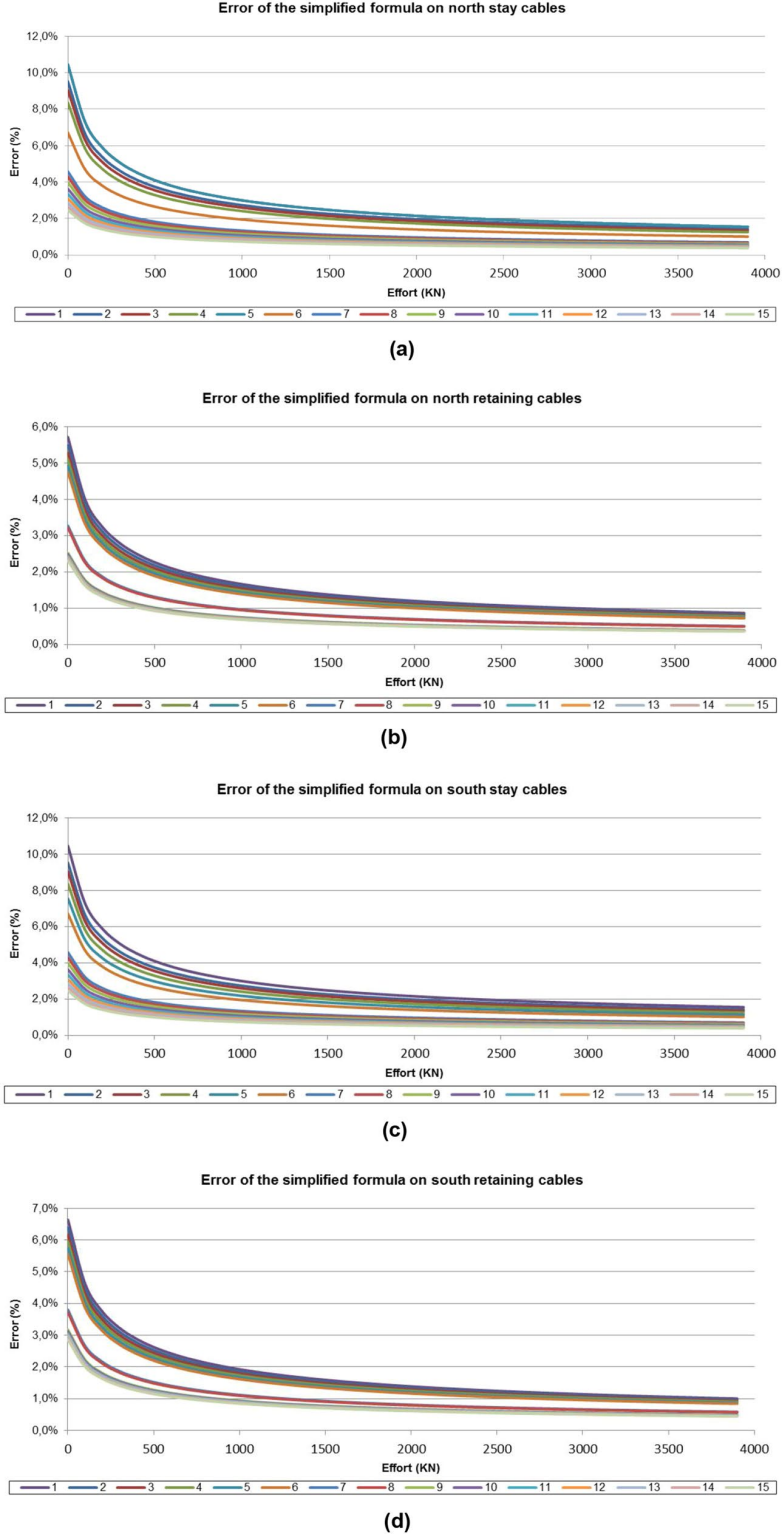
Evolution of the error committed by the simplified formulation of the vibrating wire: (**a**) north suspension cables; (**b**) north retaining cables; (**c**) south suspension cables; (**d**) south retaining cables.

The bending stiffness (E·I) in the bridge cables is negligible with respect to their axial stiffness. This fact makes the second and third sums of Eq. (3) negligible with respect to the first, resulting in Eq. (4) that relates the axial stress of the cable to its mass and frequency^19^ of oscillation. Figure 9 shows the evolution of the error committed, assuming the simplification set forth in Eq. (4), for each of the families of temporary stay cables of the Tajo River Viaduct, as a function of the stress experienced by these structural elements.

4 $$f = \frac{u}{2 \cdot L} \cdot \sqrt{\frac{T}{m}}$$

where T = Axial stress on the cable; u = Considered vibration mode; f = Vibration frequency corresponding to u-mode; E = Modulus of elasticity of the material composing the cable; I = Moment of inertia of the cable; L = Vibration length of the cable; m = Mass per linear meter of the cable.

**Figure 10:**
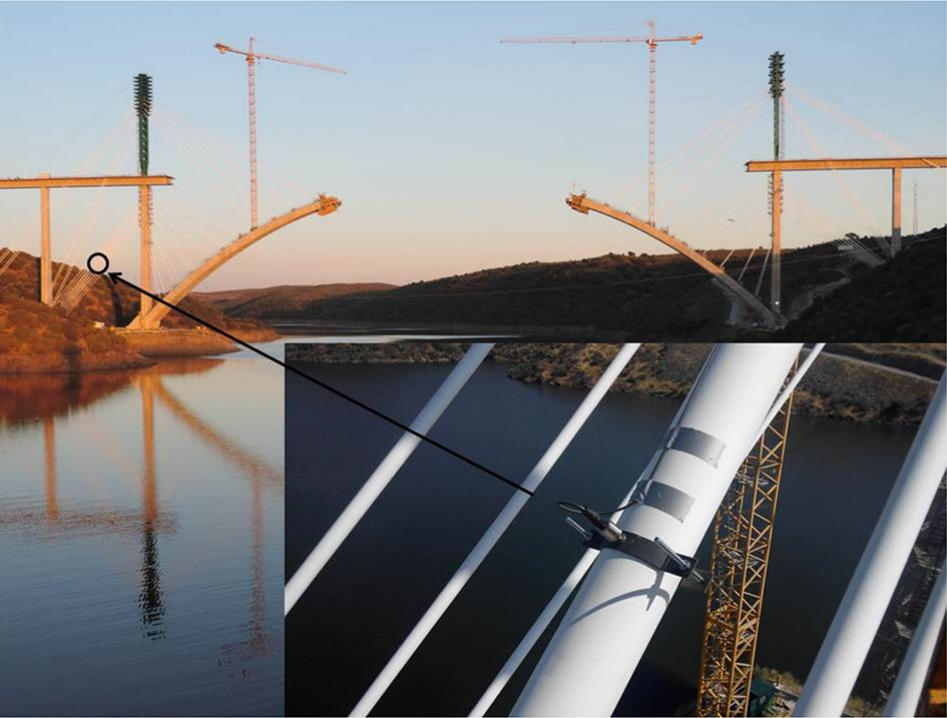
Piezoelectric accelerometer on the temporary tie of the Tajo Viaduct.

**Figure 11:**
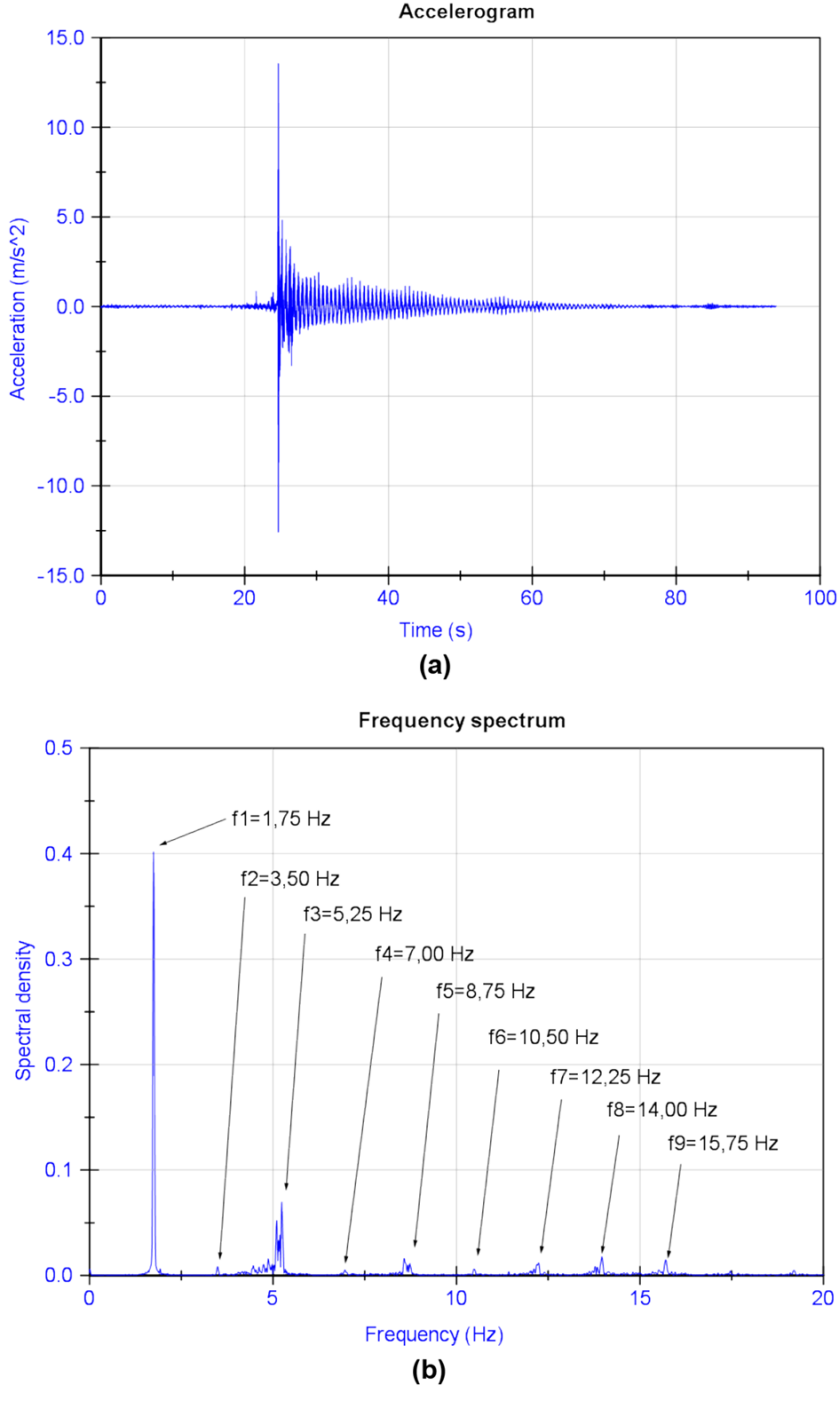
Obtaining the stresses in the fifth stay cable family from their vibration frequencies: (**a**) accelerogram; (**b**) frequency spectrum.

To carry out the weighing of the cables using the vibrating wire technique, a piezoelectric accelerometer was installed on the cable under test by means of a tool which picks up the sheath–strand assembly, excites the cable and measures the accelerations experienced by the cable during its movement in free vibration are measured. To obtain the axial stress of the cable by applying Eq. (4), it is necessary to consider the total mass moved. In the case of the temporary cables of the Tajo River Viaduct, it was necessary to consider the mass of the strands making up the cable together with the mass of the protective sheath (see Fig. 10). Another important parameter for the correct determination of the stress in bridge stay cables by means of the vibrating wire technique is the length of the stay cable. In the case of the Tajo Bridge Viaduct stay cables, this length was obtained by means of topography. The records of the acceleration experienced by the cables were analyzed by applying the Fast Fourier Transform^58–61^, obtaining the eigenfrequencies of the cables, and consequently their axial stress (see Fig. 11).

**Figure 12:**
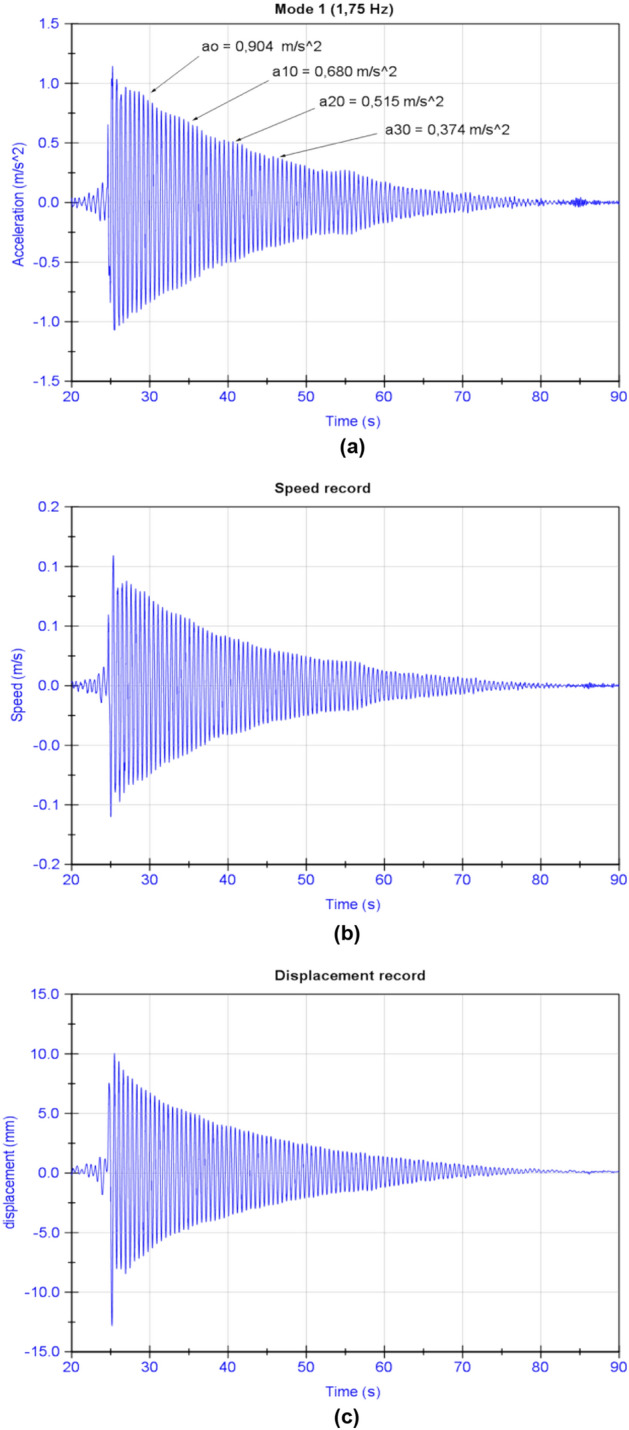
Retrieval of the damping factor of the right-hand retaining cable of the fifth family of temporary cables of the northern half arch of the Tajo River Viaduct: (**a**) recording of accelerations associated with the first mode of vibration; (**b**) speed record; c) displacement record.

Due to previous experience with the construction of similar bridges^8,60,62–64^ in which episodes of confluence between the frequency of vortex generation by the action of the wind on the structure and its vibration frequency occurred, the vibrating wire testing campaign was used to obtain the damping factor of the cables of the Tajo River Viaduct. In order to obtain the damping factor (see Fig. 12), the test consisted in subjecting the cable to an initial tension by applying a displacement to its main span and then releasing it abruptly, leaving it to oscillate in a free vibration regime. Obtaining this parameter made it possible to update the calculation model of the structure and to predict the behaviour of the stay cables in the event of possible aeroelastic phenomena. Table 1 shows the value of the damping factor obtained for the first two families of suspension and retaining cables. The damping factor of the cable is obtained from the logarithmic decrease between maximum amplitudes of its oscillation during free vibration, see Eq. (5)^65^:

5 $${\upeta } = \frac{c}{{c_{c} }} = \frac{1}{2 \cdot \pi \cdot n} \cdot ln\left( {\frac{{A_{o} }}{{A_{n} }}} \right)$$

where η = Damping factor with respect to the critical; n = Number of cycles considered for the analysis; A0 = Maximum amplitude in the initial oscillation considered for the analysis; An = Maximum amplitude in the nth oscillation considered for the analysis.

**Table 1:** Damping factor obtained for the first two families of suspension and retaining cables.

	North suspension cables	North retaining cables	South suspension cables	South retaining cables
Damping factor (%)	1.61	1.72	1.64	1.80

**Figure 13:**
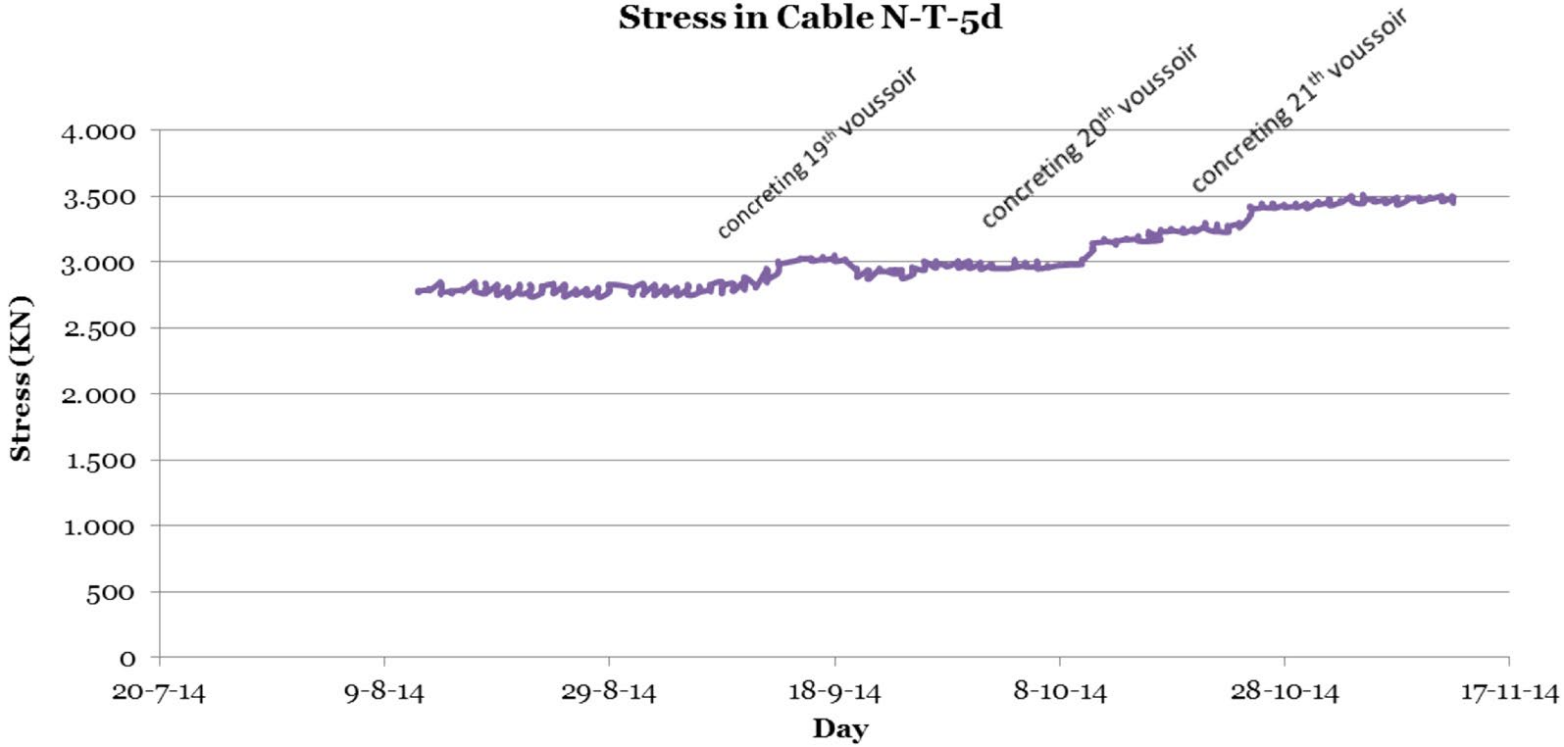
Monitoring of the evolution of the stresses in the right-hand retaining cable belonging to the fifth family of temporary cables of the northern half arch of the Tajo River Viaduct (N-T-5d) using a piezoelectric accelerometer.

Seeking a dual purpose, the authors proposed a solution that would make it possible to monitor the evolution of stresses in the cable and to characterise the possible appearance of aeroelastic phenomena caused by resonance or similar phenomena^8,60,62–64^. This solution consisted of the permanent instrumentation of the right-hand retaining cable belonging to the fifth family of temporary cables of the northern half arch of the Tajo River Viaduct (N-T-5d). For this purpose, a piezoelectric accelerometer was installed on the selected cable using a tool identical to the one used in the vibrating wire spot testing campaign, and this sensor was connected to the SMS of the Tajo River Viaduct. Figure 13 shows the monitoring of the evolution of stresses in the cable obtained from the analysis of the data provided by the piezoelectric accelerometer.

**Figure 14:**
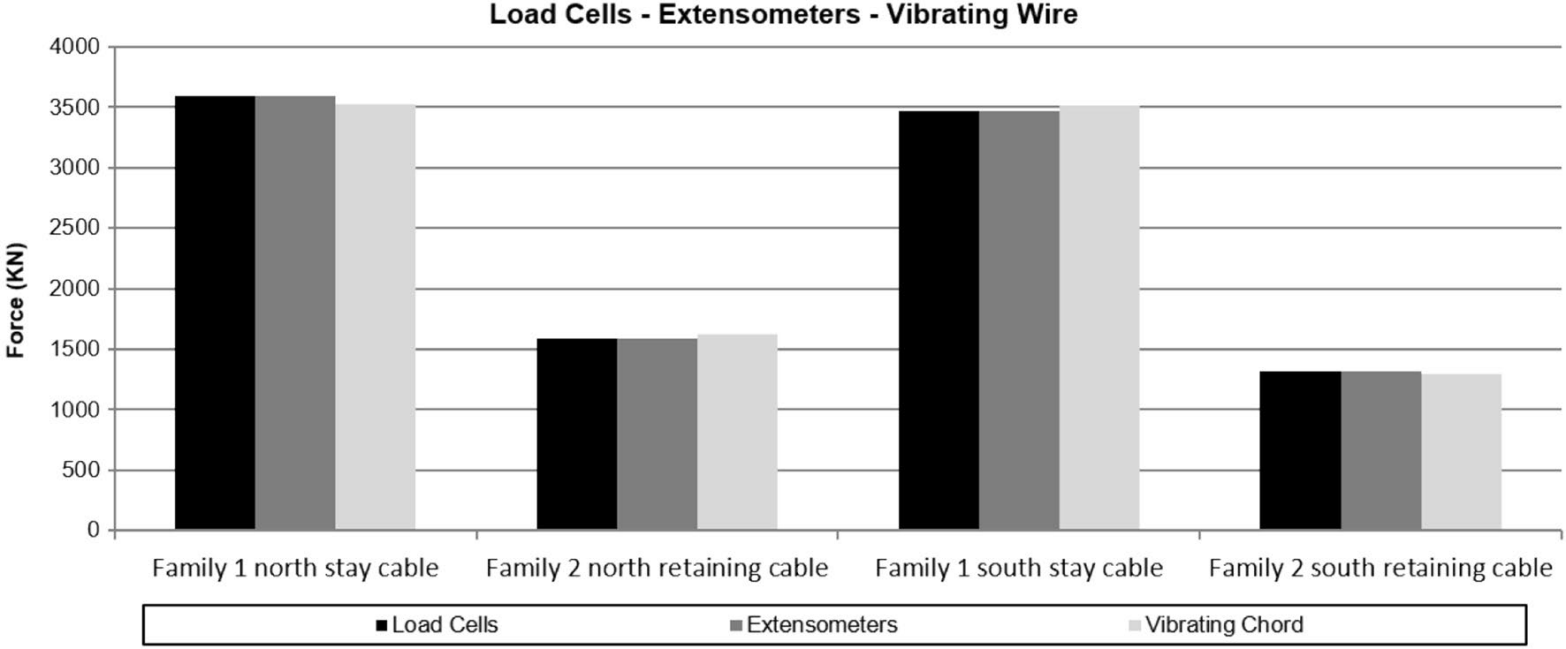
Effort facilitated by each of the structural monitoring techniques used.

The simultaneous instrumentation of the first two families of stay cables by means of load cells and unidirectional strain gauges installed in one of the strands that make up the stay cable, together with the campaign to weigh the stay cable by means of the vibrating wire technique, allowed a comparative analysis of the stress provided for each of the structural monitoring techniques. Figure 14 and Table 2 show the stress provided by each of the structural monitoring techniques used by the authors.

**Table 2:** Effort facilitated by each of the structural monitoring techniques used.

	Time stamp	Load cell (KN)	Extensometer (KN)	Vibration wire (KN)
Family 1 North suspension cable	16/05/2014 9:06	3501.4	3591.1	3525.0
Family 2 North retaining cable	16/05/2014 10:36	1632.7	1586.4	1624.7
Family 1 South suspension cable	17/05/2014 9:30	3493.0	3465.6	3511.2
Family 2 South retaining cable	17/05/2014 10:10	1299.9	1319.6	1295.2

## Lessons learned

The lessons learned during Tajo’s Viaduct monitoring through the installation of the different sensor technologies can be summarised as follows (see Table 3):

**Table 3:** Summary table for the validation of different tested technologies based on Tajo viaduct experience.

	Cost sensor	Cost readout	Long-term accuracy	Durability	Density of info
Load cells (D)	$$$	$	Very good	Very good	Low
Electrical/MEMS (D)	$	$$	Poor	Poor	Low
Vibrating wire (I)	$$	$$	Very good	Medium	High

Strain gauge load cells

- Advantages:
  o Allows measurement of the stress on the cables with an error of less than 1%.
  o Robust solution, shock-resistant, and weather-resistant solution.
- Disadvantages:
  o It represents the largest economic investment.
  o Instrumentation of heavy-duty cables involves the design of large, very heavy load cells which are difficult to handle and install on-site.
  o Installation is only possible prior to the cable installation operation.

Unidirectional strain gauges

- Advantages:
  o Technique involving the lowest economic investment.
  o Installation is possible at any stage of the construction process.
- Disadvantages:
  o Less robust solution, very sensitive to shocks and weather.
  o In the case of installation, once in service, the cable provides the stress increments on the structural element, but it is not possible to obtain the absolute value of the stress in the cable.
  o The accuracy of the measurement of stress on the cables is strongly influenced by the correct on-site installation of the device.

Unidirectional accelerometers

- Advantages:
  o Installation is possible at any stage of the construction process.
  o Allows the recording of the absolute value of the stress on the cable stress regardless of the phase of the construction process in which the sensor has been installed.
  o Allows measurement of the stress in the cables with an error of less than 1%.
- Disadvantages:
  o Technique generates a high volume of data and it is necessary to resort to spectral decomposition techniques to obtain the stress on the cable.

## Conclusions

Thanks to this work, the authors aim to give an overview of the different monitoring systems used currently used for temporary cable force monitoring techniques during bridge construction phase. For this purpose, a review of the state of the art has been carried out. An overview between load cells, unidirectional strain gauges systems and accelerometers has also been provided. All of these methods are well established and very accurate. However, each of them has its own advantages and disadvantages in terms of installation and implementation. The most promising technique under development nowadays is image-based and can be seen as complementary to these currently available methods. Today, this technique cannot provide the same level of accuracy as current methods, but it is cheap and simple to use compared to the accelerometer, i.e. further development is still needed before it can be implemented on long-span cable-stayed bridges because their behavior is not trivial. Indeed, the long-span bridges are susceptible to environmental and traffic-induced vibration.

With all these points in mind, this article is intended to serve as a basis for all work related to the world of cable monitoring during bridge construction.

## Data Availability

The data that support the findings of this study are available from the corresponding author, Gaute A., upon reasonable request.
